# An mRNA technology transfer programme and economic sustainability in health care

**DOI:** 10.2471/BLT.24.291388

**Published:** 2024-03-27

**Authors:** Devika Dutt, Mariana Mazzucato, Els Torreele

**Affiliations:** a King’s College London, Bush House NE 4.08, 30 Aldwych, WC2B 4BG, London, England.; b Institute for Innovation and Public Purpose, University College London, London, England.; c Geneva, Switzerland.

## Abstract

The World Health Organization (WHO) set up the messenger ribonucleic acid (mRNA) technology transfer programme in June 2021 with a development hub in South Africa and 15 partner vaccine producers in middle-income countries. The goal was to support the sustainable development of and access to life-saving vaccines for people in these countries as a means to enhance epidemic preparedness and global public health. This initiative aims to build resilience and strengthen local vaccine research, and development and manufacturing capacity in different regions of the world, especially those areas that could not access coronavirus disease 2019 (COVID-19) vaccines in a timely way. This paper outlines the current global vaccine market and summarizes the findings of a case study on the mRNA technology transfer programme conducted from November 2022 to May 2023. The study was guided by the vision of the WHO Council on the Economics of Health for All to build an economy for health using its four work streams of value, finance, innovation and capacity. Based on the findings of the study, we offer a mission-oriented policy framework to support the mRNA technology transfer programme as a pilot for transformative change towards an ecosystem for health innovation for the common good. Parts of this vision have already been incorporated into the governance of the mRNA technology transfer programme, while other aspects, especially the common good approach, still need to be applied to achieve the goals of the programme.

## Résumé

L'Organisation mondiale de la Santé (OMS) a lancé le Programme de transfert de la technologie de l'acide ribonucléique messager (ARNm) en juin 2021, assorti d'un centre de développement en Afrique du Sud et de 15 fabricants de vaccins partenaires dans des pays à revenu intermédiaire. L'objectif consistait à soutenir la pérennisation et l'accès à des vaccins d'importance vitale pour les populations de ces pays en vue d'améliorer la préparation aux épidémies et la santé publique mondiale. Cette initiative vise à accroître la résilience et à renforcer la recherche vaccinale locale, ainsi que les capacités de conception et de fabrication dans différentes régions du monde, en particulier dans celles qui n'ont pas pu obtenir des vaccins contre la maladie à coronavirus 2019 (COVID-19) en temps utile. Le présent document décrit l'actuel marché mondial des vaccins et résume les résultats d'une étude de cas consacrée au Programme de transfert de la technologie ARNm et menée de novembre 2022 à mai 2023. L'étude s'inspire de la vision du Conseil de l'OMS sur l'économie de la santé pour tous, qui consiste à construire une économie allant dans le sens de la santé selon quatre axes de travail: valeur, finances, innovation et capacité. En nous fondant sur les résultats de l'étude, nous proposons un cadre stratégique orienté vers un but précis: soutenir le Programme de transfert de la technologie ARNm en tant que projet pilote afin d'évoluer vers un écosystème d'innovation en matière de santé dédié au bien commun. Certains aspects de cette vision ont déjà été intégrés dans les principes de gouvernance du Programme de transfert de la technologie ARNm tandis que d'autres, en particulier l'approche liée au bien commun, doivent encore être appliqués pour atteindre les objectifs du programme.

## Resumen

La Organización Mundial de la Salud (OMS) creó el programa de transferencia de tecnología de ácido ribonucleico mensajero (ARNm) en junio de 2021 con un centro de desarrollo en Sudáfrica y 15 productores de vacunas asociados en países de ingresos medios. El objetivo era apoyar el desarrollo sostenible y el acceso a las vacunas que salvan vidas para la población de estos países como medio para mejorar la preparación ante epidemias y la salud pública mundial. Con esta iniciativa se pretende crear resiliencia y reforzar la capacidad local de investigación, desarrollo y fabricación de vacunas en distintas regiones del mundo, especialmente en aquellas áreas que no pudieron acceder oportunamente a las vacunas contra la enfermedad por coronavirus de 2019 (COVID-19). Este documento describe el actual mercado mundial de vacunas y resume las conclusiones de un estudio de caso sobre el programa de transferencia de tecnología de ARNm realizado entre noviembre de 2022 y mayo de 2023. El estudio se guió por la visión del Consejo de la OMS sobre la Economía de la Salud para Todos de crear una economía de la salud utilizando sus cuatro líneas de trabajo: valor, financiación, innovación y capacidad. A partir de las conclusiones del estudio, ofrecemos un marco político orientado a la misión para apoyar el programa de transferencia de tecnología de ARNm como piloto para un cambio transformador hacia un ecosistema de innovación sanitaria para el bien común. Algunas partes de esta visión ya se han incorporado a la gobernanza del programa de transferencia de tecnología de ARNm, mientras que otros aspectos, en especial el enfoque del bien común, aún deben aplicarse para alcanzar los objetivos del programa.

## ملخص

أنشأت منظمة الصحة العالمية (WHO) برنامجًا لنقل تكنولوجيا الحمض النووي الريبوزي المرسال (mRNA) في يونيو/حزيران 2021 مع مركز للتطوير في جنوب إفريقيا، فضلاً عن 15 شريكًا من منتجي اللقاحات في الدول ذات الدخل المتوسط. وكان الهدف هو دعم التنمية المستدامة وإتاحة الحصول على اللقاحات المنقذة لحياة الشعوب في هذه الدول، كوسيلة لتعزيز التأهب للأوبئة، والصحة العامة العالمية. تهدف هذه المبادرة إلى بناء المرونة، وتعزيز أبحاث اللقاحات المحلية، والقدرة على التطوير والتصنيع في مناطق مختلفة من العالم، وخاصة تلك المناطق التي لم تتمكن من الحصول على لقاحات مرض فيروس كورونا 2019 (كوفيد 19) في الوقت المناسب. توضح هذه الورقة سوق اللقاحات العالمية الحالية، وتلخص نتائج دراسة حالة لبرنامج نقل تكنولوجيا mRNA، تم إجراؤها في الفترة من نوفمبر/تشرين ثاني 2022 إلى مايو/أيار 2023. وقد اعتمدت الدراسة على رؤية مجلس اقتصاديات الصحة للجميع (Economics of Health for All) التابع لمنظمة الصحة العالمية، لبناء اقتصاد للصحة يستخدم مسارات عمله الأربعة المتمثلة في القيمة والتمويل والابتكار والقدرات. واستنادًا إلى نتائج الدراسة، فإننا نقدم إطار عمل سياسي موجه بالمهام، لدعم برنامج نقل تكنولوجيا mRNA كبرنامج تجريبي للتغيير التحويلي نحو نظام بيئي للابتكار الصحي من أجل الصالح العام. وقد تم بالفعل دمج أجزاء هذه الرؤية في إدارة برنامج نقل تكنولوجيا mRNA، في حين لا تزال هناك جوانب أخرى، وخاصة أسلوب الصالح العام، بحاجة إلى تطبيقها لتحقيق أهداف البرنامج.

## 摘要

2021 年 6 月世界卫生组织 (WHO) 制定了信使核糖核酸 (mRNA) 技术转让计划，在南非建立了一个开发中心，并与来自中等收入国家的 15 个疫苗生产商建立了伙伴关系。其目标在于支持这些国家以可持续发展的方式开发并帮助当地人员获得救命的疫苗，并以此作为加强流行病防范和促进全球公共卫生发展的一种手段。该措施旨在加强世界各地的抵御能力以及当地的疫苗研发和生产能力，特别是那些无法及时获得新型冠状病毒肺炎 (COVID-19) 疫苗的地区。本文概述了当前全球疫苗市场，并总结了在 2022 年 11 月至 2023 年 5 月之间实施的 mRNA 技术转让计划相关案例的研究结果。该项研究以世界卫生组织全民健康经济委员会的愿景为指导，旨在利用其价值、资金、创新和能力四个工作流构建以健康为宗旨的经济。根据研究结果，我们制定了一个任务导向型的政策框架，以支持 mRNA 技术转让计划，并将其作为试行变革措施，以建立以共同利益为目标的健康创新生态系统。该愿景的部分内容已被纳入 mRNA 技术转让计划的管治领域，同时仍需应用其他方面，特别是创造共同利益的方法，以实现该计划的目标。

## Резюме

В июне 2021 года Всемирная организация здравоохранения (ВОЗ) создала программу передачи технологии на основе матричной рибонуклеиновой кислоты (мРНК) с центром разработки в Южной Африке и 15 партнерами-производителями вакцин в страны со средним уровнем дохода. Цель заключалась в поддержке устойчивого развития производства жизненно важных вакцин и обеспечения доступа к ним для жителей этих стран в качестве средства повышения готовности к эпидемиям и укрепления глобального общественного здоровья. Эта инициатива направлена на повышение устойчивости и укрепление местного потенциала в области исследований, разработки и выпуска вакцин в различных регионах мира, особенно в тех, которые не смогли своевременно получить доступ к вакцинам против коронавирусной инфекции 2019 года (COVID-19). В этом документе приводится описание современного мирового рынка вакцин и обобщаются результаты тематического исследования программы передачи технологии на основе мРНК, проведенного с ноября 2022 года по май 2023 года. Исследование проводилось в соответствии с представлениями Совета ВОЗ в отношении экономических аспектов концепции «Здоровье для всех», направленной на создание экономики здравоохранения с использованием четырех направлений работы: ценности, финансирования, инноваций и потенциала. На основании результатов исследования предложены целеориентированные подходы для поддержки программы передачи технологии на основе мРНК как одной из первых попыток изменений, направленных на создание экосистемы инноваций в области здравоохранения, ориентированных на общее благо. Частично эти представления уже учтены в управлении программой передачи технологии на основе мРНК, в то время как другие аспекты, особенно подход на основе общего блага, еще предстоит применить для достижения целей программы.

## Introduction

The coronavirus disease 2019 (COVID-19) pandemic exposed the global imbalance in vaccine production, supply and access. Through financial, political and technical support of domestic biopharmaceutical companies, governments in high-income countries gained control and autonomy of technological innovation and production capabilities for important health technologies, including vaccines, diagnostics and treatments. As a result, these countries were able to vaccinate their populations rapidly against COVID-19. However, governments in most low- and middle-income countries could not vaccinate their populations so quickly as they did not have timely access to COVID-19 vaccines.^1^ In some cases, vaccines were not available until 2 years after the World Health Organization (WHO) declared COVID-19 a pandemic in March 2020. The reasons for these inequities are varied. However, from the public health perspective, the importance of having domestic vaccine research and development and production capacity in all regions of the world, especially for the versatile messenger ribonucleic acid (mRNA) technology, have become evident.

**Fig. 1:**
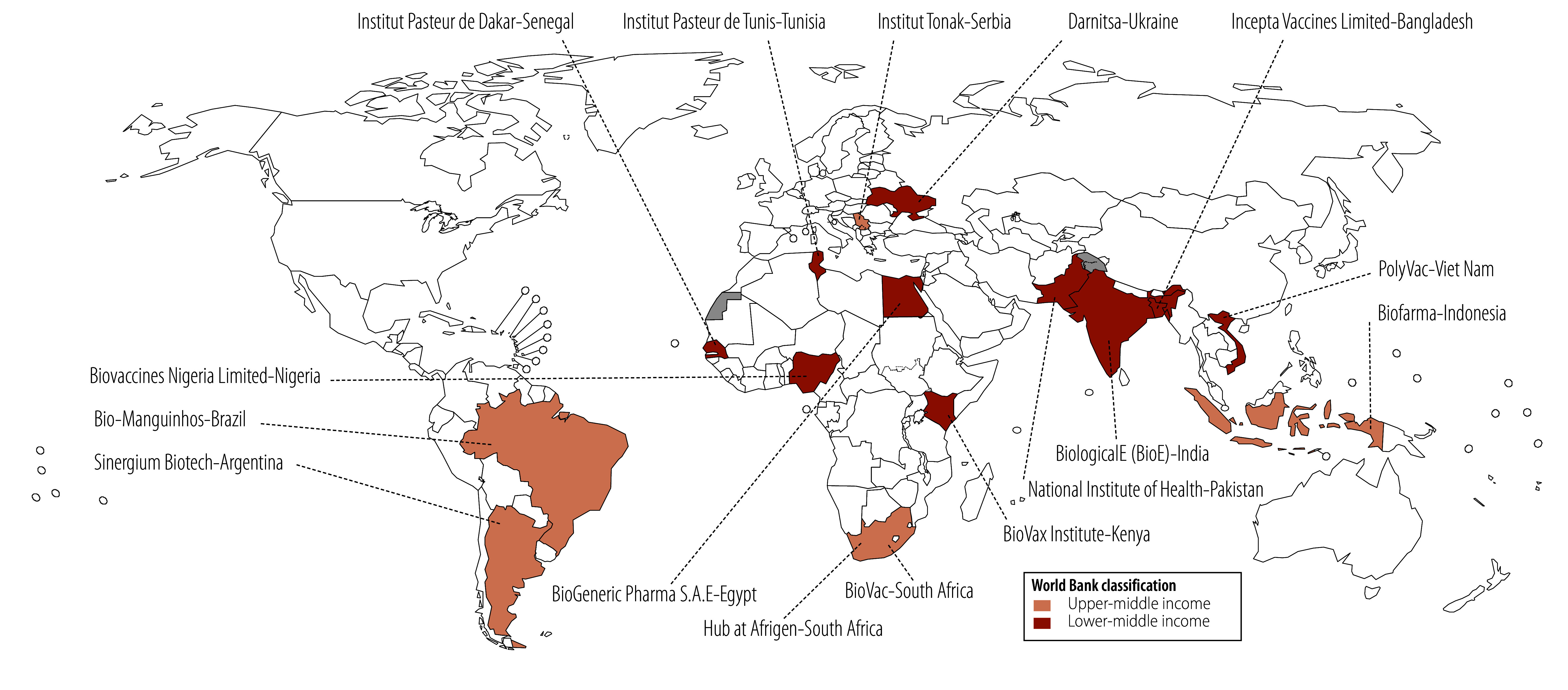
mRNA technology transfer hub and manufacturing partners

It is in this context that WHO created the mRNA technology transfer programme in mid-2021 to meet requests from low- and middle-income countries for support in developing their local vaccine manufacturing capacity and responding to the COVID-19 pandemic. The objectives of the mRNA technology transfer programme are to build resilience and sustainable capabilities for mRNA technology research and development and manufacturing to address local health needs in low- and middle-income countries. The programme was initially set up as a technology development hub at Afrigen in South Africa that would transfer the technology to partner companies in 15 middle-income countries (Fig. 1). The goal was to enable these countries to enhance their response to local health needs, build resilience for epidemic preparedness and reverse global inequities related to access to life-saving vaccines.

mRNA: messenger ribonucleic acid.

Despite its relative novelty, mRNA technology is uniquely suited for decentralized capacity-building in low- and middle-income countries. In addition to the versatility and adaptability of mRNA technology as a technology platform (with potential applicability in multiple disease areas), small- to medium-scale manufacturing infrastructure and capability can be quite easily built without the complex set-ups needed for traditional biological vaccines. Building mRNA vaccine development capabilities in the partner firms has the potential to serve not only their respective national markets, but also regional markets. In fact, most stakeholders saw this programme as addressing regional public health needs, especially to achieve a minimum viable scale of production. While the programme was set up to produce COVID-19 vaccines, new COVID-19 vaccines are not needed at scale in the near future, the earliest time by which the vaccines produced by the programme will be available. However, the development and production of AfriVac 2121 COVID-19 vaccine at Afrigen would be a validation of the successful transfer of technology, which could then be used for the development of other vaccines, such as those for influenza, dengue, tuberculosis and human immunodeficiency virus, among others.

The WHO Council on the Economics of Health for All was tasked by the WHO Director-General to reimagine economic principles, with health, well-being and equity at the centre. The Council commissioned a case study, conducted from November 2022 to May 2023, to evaluate the mRNA technology transfer programme in terms of the Council’s main themes of rethinking value, finance, innovation and capacity in the economy. This paper contextualizes the development of the programme within the global vaccine market, and summarizes the case study on the programme and its findings. The case study suggested that the programme be viewed as a collective effort among stakeholders towards resilient epidemic preparedness and response capacity for the common good,^2^ driven by collaboration between developing countries as well as pursuing the shared mission of health security, centred around equity and local resilience.^3^

## Methods

The case study was informed by literature reviews including reports of the WHO Council on the Economics of Health for All and other sources of progressive health economic thinking and global health policy, and unstructured interviews with stakeholders in the mRNA technology transfer programme. These stakeholders included staff of WHO, the Medicines Patent Pool, Afrigen (the South African vaccine manufacturing firm at the centre of the technology transfer programme), representatives of seven participating vaccine manufacturers and civil society stakeholders, among others.^2^ The focus of the discussions was how sustainability in vaccine production was being conceptualized in the mRNA technology transfer programme and whether and how the programme should be re-thought to achieve its public health objectives. The policy environment needed to facilitate the success of the technology transfer programme was also an important part of the conversations with stakeholders.

## Vaccine market landscape

The global vaccine market is segmented. Vaccines in high-income countries are supplied by global pharmaceutical corporations which can achieve substantial profit margins by charging high prices for newer vaccines and moderate and differential prices for the older routine vaccines. On the other hand, low- and middle-income countries are supplied largely by developing country vaccine manufacturers, which operate on a low-price, high-volume and low-profit-margin model, especially for older routine vaccines. The low profit margin makes substantial investment in research and development difficult. Newer vaccines are mostly only available at high prices in these markets, albeit the prices are lower than in high-income countries.^9^ In general, governments play a significant role in vaccine supply and delivery, but the support is greater in high-income countries where governments have more fiscal capacity. Vaccine markets in low- and middle-income countries are largely supported by donors, especially in countries that are eligible to receive support from GAVI, the Vaccine Alliance.^9^

Governments in high-income countries have created a policy environment that is conducive to the development and production of new vaccines by pharmaceutical companies.^10^ Notably, governments in the European Union, the United Kingdom of Great Britain and Northern Ireland, and the United States of America typically fund the high-risk early phases of research and development and invest in basic and applied research, including clinical trials. These governments also use procurement policies to ensure that pharmaceutical companies have a guaranteed market. In addition, the profits^11^ of these companies are further protected by a generous intellectual property framework that grants them broad and upstream (that is, patented by components, rather than final product) patents to privatize the results of government-supported research with no conditions attached.^12^ In other words, governments in high-income countries have many tools to shape the vaccine research and development ecosystem to deliver medical innovation and create profitable market opportunities for pharmaceutical companies.^10^^,^^13^^,^^14^ However, these government interventions are not designed for, and often get in the way of, global public health and equity, as they do not facilitate, and can impede, equitable access to affordable life-saving treatments and vaccines globally.^15^^,^^16^

Before COVID-19, the vaccine market was considered balanced in terms of market demand and supply to fulfil vaccine orders. In 2019, a total of 5.5 billion doses of vaccines were produced and purchased, representing a market value of 33 billion United States dollars (US$).^17^ However, the market is unbalanced in terms of monetary value distribution, with an estimated 68% of the market by value being in high-income countries for just 13% of the doses. Self-procuring middle-income countries, including China and India, represent 25% of the market by value for 49% of the doses. The procurement for lower-income countries subsidized by GAVI and the United Nations Children’s Fund (UNICEF) represents only 3% of the market’s monetary value for 33% of all doses.

In 2021, 16 billion doses of vaccine were produced and procured, of which 10.8 billion were for COVID-19, representing a market size of US$ 99 billion. The market for non-COVID vaccines – 5.3 billion doses worth US$ 42 billion – remained roughly unchanged.^18^ Consequently, donors such as GAVI and UNICEF drive much of the market dynamic and activities focused on fixing market failures for vaccine supply to low- and middle-income countries and rely on a handful of large-volume, low-cost producers.

Balancing supply and demand of the market does not necessarily translate into health equity and access. Important gaps remain in vaccination coverage in low- and lower-middle-income countries,^19^ even for routine vaccines that are part of WHO’s Essential Programme on Immunization and are in principle available at low cost. Vaccine inequities are greater for the newer generation, more expensive vaccines, such as the human papilloma virus, pneumococcal conjugate and rotavirus vaccines.^20^ In addition, regular shortages occur, especially for outbreak vaccines with limited and unpredictable markets. For example, a shortage of cholera vaccines occurred recently after one of only three producers decreased its vaccine production just when the frequency of cholera outbreaks was increasing.^21^ This situation results in part because it is not profitable to maintain reserve capacity for vaccines, especially for diseases that predominantly afflict people in low- and middle-income countries. Therefore, as a matter of course, the market for vaccines does not maximize vaccine coverage, nor does it serve public health well.^18^

If the mRNA technology transfer programme is to achieve its objectives, it must not replicate the market dynamics that underlie the current segmented vaccine market. Instead, the programme should establish a mission-oriented policy framework for an end-to-end ecosystem for health innovation for the common good.^4^^,^^5^ This approach would require reshaping health industry research, developing and manufacturing ecosystems for health equity, doing more than just fixing market failures, and putting the concept of the common good at the centre.^6^^–^^8^

## Reshaping research and development for health equity

The mRNA technology transfer programme was set up as a WHO-led technological capacity-building project for individual manufacturers in low- and middle-income countries.

Conversations with stakeholders did not reveal a uniform view on economic sustainability. At a minimum, donor support could be used to establish mRNA capacity in each partner company so that they can incorporate the technology as part of their operations as they continue to attract investors and compete in the market. Sustainability in that sense would mean that all, or as many as possible, partner companies could produce and supply mRNA products in an economically viable way. This view seems to underlie WHO’s sustainability work as presented at the WHO and Medicines Patent Pool mRNA meeting in Cape Town, South Africa in 2023.^22^

However, an alternative and more ambitious view of economic sustainability towards health for all would consider the collaborative network of manufacturing partners, and their mission-oriented government support, as the operational entity that delivers public health. In this view, a one-off catalytic investment in technology transfer would not be enough for sustainable development of production capacity. Instead, the continued development and sharing of technology and a collaborative research and development pipeline could serve as the core asset around which to articulate a sustainable value proposition for the development and production of epidemic countermeasures for the common good.^3^ For the mRNA technology transfer programme, this approach would mean that participating vaccine manufacturers would combine knowledge and resources (intellectual, human and financial) around a shared and collectively owned technology platform. A different governance structure would also be needed for this initiative, with equity, knowledge-sharing and regional resilience at its core. Participating countries would also be required to prioritize the strengthening of regulatory capacity, including working with WHO and international experts to clarify the most appropriate regulatory pathways for the next generation of mRNA products.

To make such an end-to-end platform for epidemic preparedness sustainable, the countries and regions hosting and supporting the platform must design ex-ante conducive policies for the platform to achieve its goals.^7^ Such policies should apply the vision laid out by the WHO Council on the Economics of Health for All to build an economy for health,^23^ using its four work streams of value,^24^ finance,^25^ innovation^26^ and capacity^27^ as guidance. This approach focuses on how value in health is measured, produced and distributed across the economy, and how innovation is governed to provide low- and middle-income countries with the ability to invest in initiatives such as the hub, and the capacity, both public and private, to make it happen.

### Valuing what matters

Markets value goods and services in terms of prices, but this system is not a good indicator of the value of public goods such as health.^7^^,^^28^^,^^29^ For instance, the value of a vaccine includes individual health benefits and broader socioeconomic and indirect impact(s) that the vaccine or vaccination might have, as reflected in WHO’s full vaccine value assessment, an analysis to inform priority-setting for investment in and uptake of vaccines.^30^ For epidemic preparedness and response in particular, the capacity of countries or regions to rapidly develop and make available health technologies to control outbreaks when and where they occur is an important asset for health security. This capacity needs to be valued as such, even if it is not profitable for individual companies.^31^ Other drivers of local resilience and equity are technological capability and autonomy to develop innovative solutions to address local health needs.^32^ The mRNA technology transfer programme would need to be reconfigured such that these drivers of public health, reliance and health security are valued, even if the traditional metrics of price and profits dictate otherwise.

To this end, the programme should measure success not just by the revenue streams generated by the partner companies, but by a mix of factors that includes: collaboration between firms and health-care providers to co-create vaccine candidates for an emerging health threat; ability to produce vaccines and related products and obtain national regulatory approval within a reasonable time frame; establishment of multiple small- to medium-scale production units that can produce epidemic countermeasures at acceptable cost-of-goods, and are ready for activation when needed; ability to establish reserve capacity that can be rapidly activated in case of need or to supply stockpiles; and increased access to relevant vaccines, and adequate and timely coverage.

### Financing what is valued

If both the societal value of vaccines (based on the full vaccine value assessment) and the strategic value of an end-to-end system for epidemic preparedness and resilience are recognized as goals of the mRNA technology transfer programme, appropriate channels need to be used to mobilize the required financing.

The programme currently receives its funding from donors mostly from high-income countries as part of their development cooperation (Table 1). As of May 2023, the programme has US$ 128.9 million in commitments, with about three quarters allocated to the South African Consortium (Hub) around Afrigen, BioVac and the South African Medical Research Council, and a quarter to the partner companies.

**Table 1:** Funding committed for the mRNA technology transfer programme, by donor country, May 2023

Funder	Amount, in million US$
French government	54.4
Canadian government	33.9
European Commission	12.0
German government	6.6
African Union	7.0
South African government	4.5
Belgian government	4.3
Norwegian government	4.5
Other	1.7
**Total**	**128.9**

mRNA: messenger ribonucleic acid; US$: United States dollars.

Equipping the whole network of manufacturing partners with state-of-the-art infrastructure for efficient small- to medium-scale production will require further investments for the manufacturers, through additional donor funding and domestic finance, especially if governments can access capital affordably. However, not all partners will be able to individually raise the finance needed, given the uncertainty about whether the market for a vaccine candidate would exist and the lack of procurement guarantees from governments or multilateral organizations. Current budget estimates are modest to set up the needed infrastructure and capability, develop mRNA technology and transfer it to the 15–20 manufacturers and expect them to be sustainable.

Therefore, greater financing needs to be secured for the programme from international financial institutions, regional development banks and potentially private financial sources.^33^^–^^35^ However, the funds to finance an end-to-end approach for health security would have to be carefully selected and directed with appropriate conditions.^36^ Unlike typical public–private partnership funding, the arrangement should not only minimize risk to the investments of private sector partners and investors, but also ensure the collective effort is structured around shared objectives of all stakeholders to achieve health security, resilience and sustainability.

### Governing innovation

The shortcomings of the current commercial health innovation system to serve public health objectives are well documented,^26^ and are particularly apparent for epidemic preparedness and response.^37^^,^^38^ With intellectual property monopolies as the main mechanism to maximize financial returns, research and development priorities are geared to market opportunities rather than health needs. This model allows billions of dollars in profits for large pharmaceutical companies while not disclosing the know-how and technologies that could transform pandemic preparedness and response in low- and middle-income countries.^39^ At the same time, new health products command increasingly high prices, even though most such goods show little or no clinical benefit compared with products that already exist.^29^ These dynamics also result in insufficient research and development investment in treatments and vaccines for diseases that affect people in low- and middle-income countries, where the population does not have the means to pay high prices.^40^

The mRNA technology transfer programme should be designed as a collectively owned research and technology platform for epidemic preparedness and response which is managed for the common good.^3^^,^^31^ An appropriate legal structure and organizational form should be designed in which the shared technology platform and research and development portfolio are considered important common-good assets for public health. Access and user rights should be defined around this platform and portfolio and linked to commitments to continued investments in sustainability and equitable access to the resulting health products when and where needed.^2^

Success of the mRNA technology transfer programme requires the implementation of measures to ensure freedom to operate without intellectual property constraints in developing, manufacturing, commercializing (including for export) and using health products produced with mRNA technology.^3^ As such, individual government action, or regional and/or global approaches, may be required, including the use of legal mechanisms under international law if advocacy and good-faith negotiations fail to secure essential tools for public health.

### Building government capacity

Countries that host a local manufacturer participating in the mRNA technology transfer programme have in principle committed to support the manufacturing partners. Concrete ways to support the programme include: (i) ensuring that the country’s intellectual property laws include flexibilities within the Trade-Related Aspects of Intellectual Property Rights to overcome any intellectual property barriers to protecting public health, and the willingness to use these laws if needed to achieve the goals of the mRNA technology transfer programme; (ii) strengthening regulatory oversight and authorization capacity and the ability to make judgements about benefits or risks based on the local context – or building international collaboration to support this function; (iii) ensuring the timely adoption of evidence-based vaccination guidelines, and closely coordinating with vaccine procurement (locally and internationally); (iv) investing in building local research and development capacity and creating opportunities for research driven by local health needs; (v) promoting national and regional collaboration and open science initiatives; and (vi) mobilizing domestic and international finance to support the mRNA technology transfer programme as investment in both local and global health security and equity.

## Conclusion

The mRNA technology transfer programme is a timely, important and ambitious project to shift the balance of global vaccine production so that researchers and developers in low- and middle-income countries can produce life-saving health technologies and provide equitable access to them in a timely way. Establishing resilient global health security infrastructure and capability and supporting the freedom for research, development and manufacture of vital health technologies are essential building blocks towards that goal. However, ensuring success requires rethinking the definition of sustainability and reshaping the health–industry system for health equity. This change involves moving beyond the concept of competitive markets and market-fixing to enable individual producers to thrive as businesses. Therefore, a new narrative and value proposition must be adopted that focuses on mission-oriented economic sustainability for health from a country, regional and global perspective. To build a new narrative and achieve the desired outcomes for health, a range of inputs, policies and operational mechanisms need to be considered which include access to suitable technologies and know-how, adequate financing, skilled human resources, and collaboration and coordination between developing countries. While parts of this overall vision have started to be incorporated into the evolving design and operationalization of the mRNA technology transfer programme, other aspects – especially the common good approach to health security through technological capability and freedom to operate – have yet to be seriously applied. Stronger leadership and a global public health vision from the partners in low- and middle-income countries and their governments are needed to move from a vertical technical assistance project piloted by WHO and Medicines Patent Pool to a truly and locally owned collaborative health security effort for the common good, rooted in regional resilience and autonomy.
